# Multisession Anodal tDCS Protocol Improves Motor System Function in an Aging Population

**DOI:** 10.1155/2016/5961362

**Published:** 2016-01-10

**Authors:** G. Dumel, M.-E. Bourassa, M. Desjardins, N. Voarino, C. Charlebois-Plante, J. Doyon, Louis De Beaumont

**Affiliations:** ^1^Centre de Recherche de l'Hôpital du Sacré-Cœur de Montréal, 5400 boulevard Gouin Ouest, Montréal, QC, Canada H4J 1C5; ^2^Département de Psychologie, Université du Québec à Montréal, 100 rue Sherbrooke Ouest, Montréal, QC, Canada H2X 3P2; ^3^Unité de Neuroimagerie Fonctionnelle, Centre de Recherche de l'Institut de Gériatrie de Montréal, 4545 chemin Queen-Mary, Montréal, QC, Canada H3W 1W4; ^4^Département de Psychologie, Université de Montréal, Montréal, QC, Canada H3C 3J7; ^5^Département de Psychologie, Université du Québec à Trois-Rivières, 3600 rue Sainte-Marguerite, Trois-Rivières, QC, Canada G8Z 1X3

## Abstract

*Objectives*. The primary objective of this study was to investigate the effects of five consecutive, daily 20-minute sessions of M1 a-tDCS on motor learning in healthy, cognitively intact, aging adults.* Design*. A total of 23 participants (51 to 69 years old) performed five consecutive, daily 20-minute sessions of a serial reaction time task (SRT task) concomitant with either anodal (*n* = 12) or sham (*n* = 11) M1 a-tDCS.* Results*. We found a significant group × training sessions interaction, indicating that whereas aging adults in the sham group exhibited little-to-no sequence-specific learning improvements beyond the first day of training, reproducible improvements in the ability to learn new motor sequences over 5 consecutive sessions were the net result in age-equivalent participants from the M1 a-tDCS group. A significant main effect of group on sequence-specific learning revealed greater motor learning for the M1 a-tDCS group when the five learning sessions were averaged.* Conclusion*. These findings raise into prominence the utility of multisession anodal TDCS protocols in combination with motor training to help prevent/alleviate age-associated motor function decline.

## 1. Introduction

Transcranial direct current stimulation (tDCS) is a noninvasive technique of cortical brain neuromodulation, which uses constant, low intensity direct current delivered to the brain area of interest via electrodes on the scalp [1, 2]. The application of such current influences transmembrane neuronal potentials and covertly modifies the level of neuronal excitability via activation of cerebral plasticity mechanisms [2–5]. Depending on the polarity of the active electrode applied to the brain, this technique can either increase (anodal) or decrease (cathodal) cortical excitability of the targeted region [5, 6].

The major interest in these tDCS aftereffects is that tDCS modulates cortical excitability and brain function [7]. Indeed, anodal tDCS (a-tDCS) has been applied over many cortical areas in an attempt to increase their function. For instance, studies showed that a-tDCS over the dorsolateral prefrontal cortex can enhance language processing [8] and working memory [9] or increase pain empathy [10] in healthy subjects. A-tDCS has also been tested over the dorsomedial frontal cortex during the execution of a stop-signal task and was associated with inhibitory control improvements in healthy participants [11]. However, the utility of a-tDCS is best validated in studies aiming to modulate primary motor cortex (M1) excitability and associated motor functions. M1 is highly involved in motor execution and learning as well as in procedural memory formation including the consolidation of motor skills [7, 12–14].

It is generally agreed that a-tDCS-dependent behavioral gains are optimized with concurrent behavioral training [2, 15–18]. For example, during a serial reaction time task (SRT task) classically used to study implicit motor sequence learning, the M1, premotor, or prefrontal cortices were stimulated contralaterally to the performing hand [19]. Relative to sham tDCS stimulation, a single-session a-tDCS stimulation of M1 resulted in increased SRT task performance, whereas stimulation of the premotor and prefrontal cortices had no effect. These findings suggest that a-tDCS concomitant to SRT task performance accentuates implicit motor learning effects [19].

In addition, M1 a-tDCS is well adapted for motor rehabilitation as it can be safely applied for up to 30 minutes when tested with current charges up to 2 mA at a current density of 0.04 mA/cm^2^ [20]. Single-session M1 a-tDCS has been found to exert significant beneficial effects on motor function in clinical populations including chronic stroke and traumatic brain injury patients [3, 20–22]. Improved motor execution speed is the typical net result of such M1 a-tDCS motor training protocol, whether obtained from the paretic hand of stroke patients or in healthy controls [3, 20–22]. However, clinical utility of single-session tDCS interventions is restricted, as stimulation aftereffects are generally short-lived and not robustly replicated across studies [23]. Multisession protocols, however, have proven to induce more reliable effects on both cortical excitability and behavioral gains and these beneficial aftereffects tend to outlast a-tDCS intervention [18, 24, 25]. Accordingly, a recent study found that a-tDCS given continuously at 2 mA for 20 minutes induced changes in M1 excitability that lasted for at least 2 hours, with further cumulative increases in excitability when sessions were repeated on a daily basis over a 5-day period [26]. In the same vein, a significant cumulative increase in cortical excitability was found with the application of a-tDCS over M1 for five consecutive weekdays [27]. In addition, a recent study conducted in healthy controls applied a-tDCS over M1 while subjects acquired a sequential finger tapping task over three consecutive days. It was found that the sequential finger tapping task benefited significantly from a-tDCS during learning relative to controls assigned to the sham stimulation group [28]. Furthermore, in young healthy controls, five daily, consecutive, 20-minute sessions of M1 a-tDCS combined with a motor learning task were shown to induce reproducible, online task performance improvements that were found to persist beyond three months after intervention [15].

Knowing that tDCS mechanisms of action involve neuronal plasticity, age-associated decline of synaptic efficacy would be expected to influence tDCS aftereffects. Previous TMS studies have highlighted the significant decline of M1 neuronal plasticity in the aging population [5, 29]. Age-related brain plasticity reduction is of critical clinical significance as it has abundantly been associated with cognitive decline and increased prevalence of neurodegenerative diseases [29, 30]. Yet, while numerous studies have documented the beneficial effects of a-tDCS on brain function in younger adults, evidence supporting the fate of a-tDCS protocols in ameliorating brain functioning in older individuals remains limited. To date, single-session a-tDCS has been associated with significant improvements on picture naming (after a-tDCS to the left inferior frontal cortex; [31]), working memory (following a-tDCS to the prefrontal cortex; [32]), and object-location learning tasks (after a-tDCS to the right temporoparietal cortex; [33]). A study by Meinzer and colleagues [25] also showed that a single session of a-tDCS administered to the left inferior frontal gyrus had transiently reversed age-related semantic fluency decline. Interestingly, a significant improvement in complex motor skill acquisition [34], mimic activities of daily living [35], and visuomotor adaptation [36] has been reported after a single session of M1 a-tDCS in old individuals. Yet, an interesting and unexplored application of a-tDCS would be to validate whether further functional gains could be associated with the application of multisession a-tDCS protocols in an attempt to alleviate the known deleterious impact of aging on cognitive function.

Here, we tested whether concomitant application of a-tDCS on M1 while performing five daily, 20-minute sessions of an implicit motor learning task would lead to greater task improvements in an aging population when contrasted with that of a sham stimulation group. We hypothesized that aging individuals receiving M1 a-tDCS stimulations over five consecutive days would exhibit significantly greater implicit motor learning improvements in comparison to a matched control group assigned to the sham intervention.

## 2. Methods

### 2.1. Participants

All 23 participants (61 ± 4.61 years old; range 51 to 69 years, 12 women) were healthy, right-handed elderly adults recruited via newspaper ads. Participants were included if they met all of the following criteria: no significant neurological history (e.g., traumatic brain injury, stroke, encephalopathy, and seizure disorder); no history of alcohol and/or substance abuse; no psychiatric illness or learning disability. None of them reported using centrally acting drugs, having movement restriction or pain in their right arm or hand, or regularly practicing any activity that involved repeating sequential finger movements (e.g., playing a musical instrument). Participants were also screened for cognitive impairment and depression using the Mini-Mental State Examination (MMSE; [37]) and the Beck Depression Inventory II (BDI-II; [38]) with cut-offs of 26 and 13, respectively. Subjects were asked not to drink coffee 4 hours before the start of each session. The study was approved by the Research Ethics Committee of the* Hôpital du Sacré-Cœur de Montréal* and all participants provided written informed consent before testing. Participants received a financial compensation for their participation.

Participants were randomly assigned to one of two groups: an anodal tDCS group (*n* = 12) and a sham stimulation group (*n* = 11). The two groups were closely matched in terms of their gender distribution (*t*(21) = .048; *P* = .827; Cohen's* d* = 0.083), age (*t*(21) = .269; *P* = .791; Cohen's* d* = 0.095), and level of education (*t*(21) = .915; *P* = .471; Cohen's* d* = 0.131). None of the participants presented any signs of depression (BDI-II scores ≤ 13, 0–13 standardized cut-off corresponding to minimal depression) or cognitive impairment (MMSE ≥ 27). Refer to Table 1 for more details.

Given the known effects of sleep on learning, the subjects' sleep quality on the night preceding testing was assessed at the beginning of each of the five sessions of the study using a custom 3-item questionnaire. Participants were asked to evaluate the quality of their sleep (on a scale from very bad to very good sleep), their mood when waking up (on a scale ranging from very tense to very calm), and their level of vigilance when waking up (on a scale ranging from very tired to very awake) by drawing a line at the appropriate place on a 10-centimeter scale. The total score was reported on 30 points, where each centimeter corresponded to a single point. The average completion time was 3 minutes. Averaged sleep quality of the night before testing, including each of the five study sessions, was equivalent across groups (*t*(21) = .732; *P* = .545; Cohen's* d* = 0.14). Refer to Table 2 for more details.

### 2.2. Experimental Procedures

**Figure 1:**
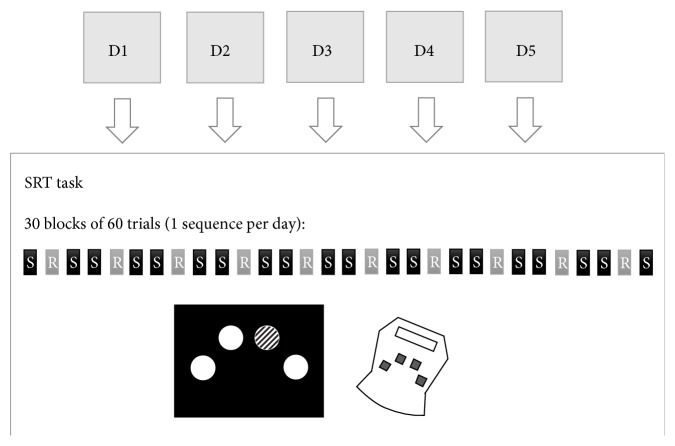
Study design and SRT task paradigm, stimuli, and keyboards. The five grey squares, D1 to D5, refer the five days of training. Grey rectangles containing the letter “R” refer to random blocks and the black rectangles containing the letter “S” refer to sequence blocks. The unscaled schematic representation of the stimuli displayed on the computer screen and the keyboard used to perform the SRT task are depicted. The yellow circle used as the GO signal is displayed here as a striped circle.

The experiment consisted of five testing sessions conducted over a period of five consecutive days (Figure 1). Each of the five sessions consisted of a 20-minute tDCS session (anodal or sham) concomitant with the execution of a modified SRT task adapted for a concurrent multisession protocol. Sessions took place between 8 a.m. and 5 p.m. and were separated by 24 h. The time of day of testing was kept constant throughout the five sessions and was equivalent between both groups. Each session lasted about 40 minutes.

### 2.3. Transcranial Direct Current Stimulation Protocol

A-tDCS was delivered through two saline-soaked sponge electrodes (7.5 cm × 6 cm) connected to a constant direct current stimulator (HDCKit, Newronika, Milan, Italy). We used a bipolar electrode montage with a 2 mA direct current flowing from an anode positioned over the left M1 to a reference electrode positioned on the contralateral supraorbital area [6]. For precise and individualized localization, the left M1 hand area was identified in all subjects using transcranial magnetic stimulation. In the anodal group, the stimulation was applied continuously for 20 minutes each day. By contrast, the same installation was used in the sham group, yet the current was interrupted after having completed the initial 30-second ramp-up. Only the investigator was aware of the type of stimulation (anodal or sham).

### 2.4. SRT Task

During tDCS application, participants performed a custom SRT task running on MatLab (version R2012b; The MathWorks, Natick, MA) and designed to measure implicit motor sequence learning [39, 40]. Each trial consisted of one filled yellow circle and 3 white circles of equal size (3.6 cm diameter), positioned at an equal distance in an inverted U shape (Figure 1). The position of the cue (yellow circle) varied across trials among the four possible locations and indicated the correct key to press. Participants were instructed to respond as fast and as accurately as possible to the position of the yellow circle by pressing the corresponding key on the game board (model G13; Logitec, Lausanne, Switzerland) with the predetermined fingers of the right hand (index for lower-left key, middle finger for upper-left key, ring finger for upper-right key, and little finger for lower-right key). Participants were instructed to perform the task only with their dominant hand and to keep the appropriate finger on each predetermined key at all times. Participants performed a total of 30 blocks separated by 15-second pauses, including 10 random (R) and 20 sequence (S) blocks of trials (Figure 1). Each block included 60 trials, each yellow circle (trial) remaining on the screen until a key press was made (correct or incorrect) and being immediately replaced by the next trial. The 20 sequence blocks consisted of five presentations of the same 12-item sequence. In order to assure that motor sequence learning remained implicit over five consecutive sessions, distinct but equivalent 12-item sequences were presented on each of the five tDCS sessions (*Session 1*: 1-2-4-3-1-3-2-1-4-2-3-4;* Session 2*: 2-3-2-4-1-3-1-4-3-4-2-1;* Session 3*: 4-3-2-4-2-3-1-2-1-4-1-3;* Session 4*: 2-4-3-2-3-1-4-1-2-1-3-4;* Session 5*: 1-2-4-1-4-2-1-3-2-3-4-3), the order of the sequence on each session being counterbalanced between subjects. The first three sequences were taken from Reber & Squire [41], while the last two were created in accordance with the criteria used by these authors. Thereby, each series contained three repetitions of each of the four possible cue locations and one occurrence of each of the 12 possible transitions between locations (e.g., 12, 13, 14, 21, and 23). Moreover, in order to limit the similarity between sequences, each transition between the 4 locations (e.g., 4-3-2-3) never repeated itself through the five sequences. The 10 random blocks were inserted among the sequence blocks. The order of presentation of the two types of blocks was the following: sequence block–random block–sequence block, repeated ten times. Each session began with a random practice block (60 trials). Refer to Figure 1 for a graphical presentation of the SRT task paradigm.

### 2.5. Measuring Motor Performance and Learning

Response time (RT) was defined as the time interval between stimulus presentation and the key press response. Motor performance corresponds to the averaged RT for sequence and random blocks independently. Sequence-specific learning (percent change in RT) per day of training was computed as follows: ((mean RT of random blocks − mean RT of sequence blocks)/mean RT of random blocks) × 100% per day. This measure allows dissecting sequence-specific learning while controlling for familiarity with the task procedure for any given day of training.

### 2.6. Statistical Analysis

All values are expressed as means ± SD. Demographic and motor performance at the SRT task were subjected to standard descriptive statistics, Student's *t*-tests, and chi-square where appropriate. Similarly, sequence-specific learning percent at the SRT task were subjected to a 2 (groups) × 5 (training sessions) mixed ANCOVA, with age, gender, and level of education as covariates of no interest. False discovery rate (FDR) corrections for multiple comparisons were also applied.

## 3. Results

### 3.1. Motor Performance

**Figure 2:**
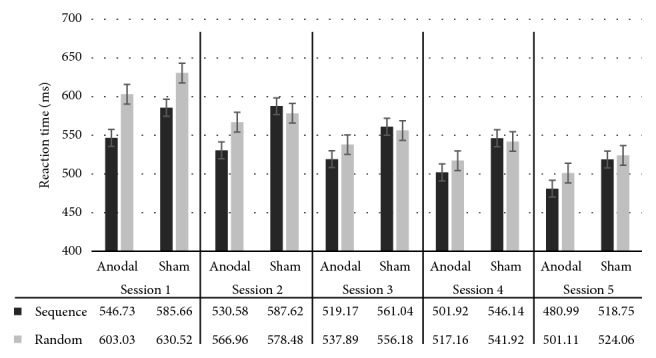
Mean RT sequence and random blocks (ms) per group and per day.

We computed RT changes in both sequence and random blocks across training days. As expected, RT significantly improved in both groups as a function of training sessions (*F*_(1,21)_ = 142.70; *P* < .0001; *η*_*p*_^2^ = 0.872). In addition, aging participants from the a-tDCS group were significantly faster at executing sequence blocks from sessions 1 and 5 (*F*_(1,21)_ = 5.63; *P* < .03; *η*_*p*_^2^ = 0.211) than participants in the sham group. However, the training sessions × group interaction on sequence blocks did not reach significance (*F*_(1,21)_ = 0.011; *P* > .05; *η*_*p*_^2^ = 0.001), suggesting that the pattern of RT improvement across training sessions was comparable between groups (i.e, both groups showed a linear RT improvement pattern from session 1 to session 5) (see Figure 2).

### 3.2. Implicit Motor Learning

**Figure 3:**
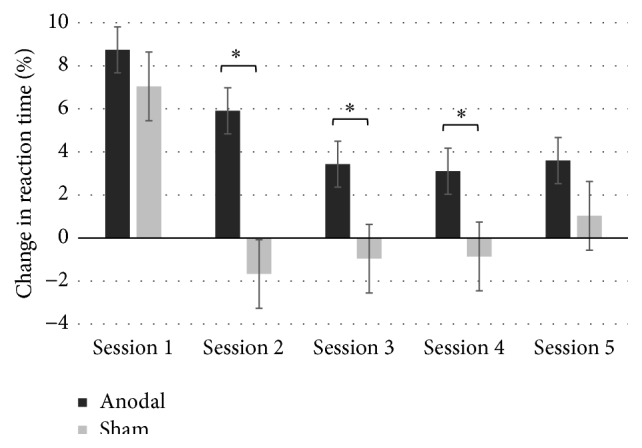
Mean sequence-specific learning (percent change in RT) per group and per session.

**Figure 4:**
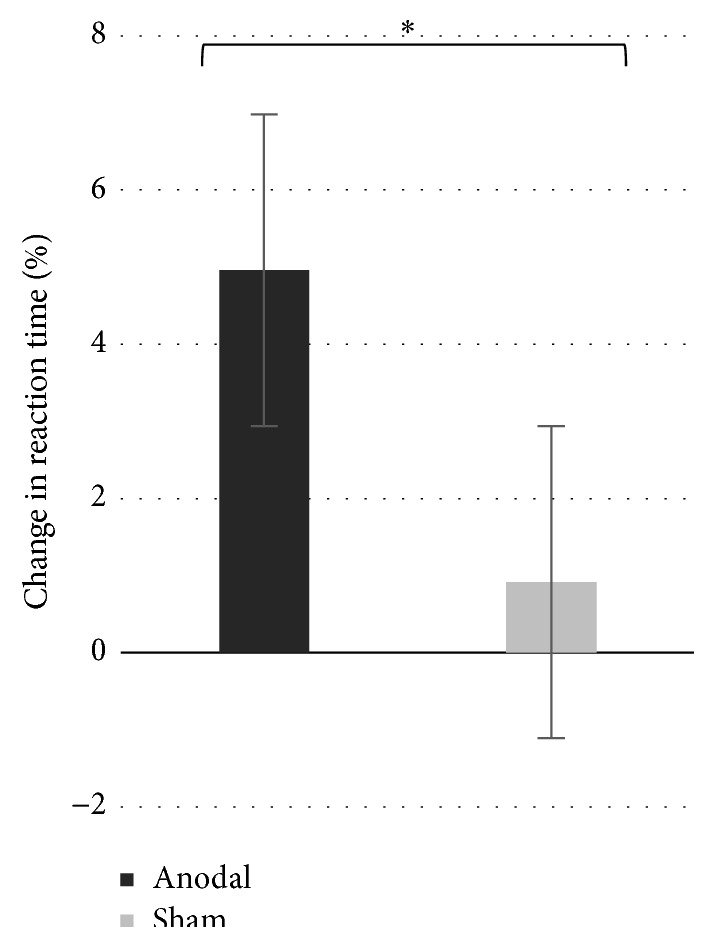
Mean sequence-specific learning (percent change in RT) across training sessions per group.

As expected, an ANCOVA revealed a significant group × training sessions interaction on sequence-specific learning when percent change in reaction times ((mean R blocks − mean S blocks)/mean R blocks) × 100% per day was collected for each participant, with age, gender, and level of education as covariates of interest (*F*_(1,21)_ = 2.61; *P* < .05; *η*_*p*_^2^ = 0.112). This finding indicates that the sequence-specific learning pattern differed across groups. We also found a significant main effect of group on sequence-specific learning (*F*_(1,21)_ = 5.28; *P* < .05; *η*_*p*_^2^ = .0228), indicating that participants from the a-tDCS group exhibited significantly greater sequence-specific learning than sham tDCS counterparts when the five learning sessions were averaged (Figure 4). Importantly and as hypothesized, we also found a significant main effect of training sessions (*F*_(1,21)_ = 17.1; *P* < .001; *η*_*p*_^2^ = 0.415), indicating that the magnitude of sequence-specific learning significantly differed across training sessions when performance data from both groups were combined. We then computed contrast analyses, FDR corrected for multiple comparisons, on sequence-specific learning (percent change) for each session independently and found the following between-groups effects: [*Session 1* (*t*(21) = .681; *P* = .504; Cohen's* d* = 0.29);* Session 2* (*t*(21) = 3.502; *P* < .05; Cohen's* d* = 1.28);* Session 3* (*t*(21) = 2.409; *P* < .05; Cohen's* d* = 1.17);* Session 4* (*t*(21) = 2.105; *P* < .05; Cohen's* d* = 0.97); and* Session 5* (*t*(21) = 1.470; *P* = .156; Cohen's* d* = 0.63)] (refer to Figure 3 for a graphical representation of mean % change in RT across groups for each session), suggesting that the M1 a-tDCS group exhibited significant sequence-specific learning improvements relative to the sham group on days 2, 3, and 4. As clearly depicted in Figure 3, the nonsignificant between-groups difference at day 5 was due to a slight regain of sequence-specific learning in the sham group, while sequence-specific learning improvement in the M1 a-tDCS group was comparable to that of the three previous training sessions.

### 3.3. Accuracy

There was no group difference in overall mean response accuracy [*sequence blocks t*(21) = −.158; *P* = .876; Cohen's* d* = 0.06;* random blocks t*(21) = .511; *P* = .615; Cohen's* d* = 0.21] as well as across training sessions, either for sequence or random blocks (refer to Table 3 for more details).

## 4. Discussion

The primary objective of this study was to investigate the effects of five consecutive, daily 20-minute sessions of M1 a-tDCS on motor learning in healthy, cognitively intact, older adults. The current findings reveal that, relative to sham tDCS, the application of M1 a-tDCS concomitant with the execution of a SRT task significantly enhanced implicit motor learning in the aging brain. This finding obtained when probing the aging brain is consistent with the young adult literature on the efficacy of M1 a-tDCS to improve motor learning [15–17, 19, 28, 42–45]. In addition, results from the present study support the added benefits of a multisession M1 a-tDCS intervention protocol to enable further improvements at an implicit motor learning task involving distinct motor sequence over five consecutive days [15, 28, 46]. Knowing that our adapted SRT task introduced a new, 12-item sequence on each testing session, multisession M1 a-tDCS aftereffects may have facilitated procedural consolidation so as to further improve implicit motor learning gains over consecutive stimulation sessions. In a previous study conducted with young adults, a five consecutive, daily 20-minute M1 a-tDCS protocol, applied during rotary pursuit task training, allowed significant, continuous explicit motor learning improvements throughout the training sessions. In the present study, we found that while older adults in the sham group exhibited little-to-no sequence-specific learning improvements beyond the first day of training, sustained improvements in the ability to learn new motor sequences were maintained throughout the five consecutive sessions in age-equivalent participants from the a-tDCS group. Thus, the present study extends previous findings as it shows that implicit motor learning also benefits from multisession M1 a-tDCS effects.

Interestingly, sequence-specific learning at session 1 was not significantly different across groups. As depicted in Figure 2, sequence-specific learning at day 1 reached nearly 8.5% in the a-tDCS group, while that of the sham group was at 7%. Our findings contrast with a previous report in which explicit motor skill acquisition improvements were significantly greater in older adults who performed a single training session with adjuvant M1 a-tDCS relative to age-equivalent sham controls [34] in older adults. Although conjectural, these results discrepancies indicate that enhanced explicit motor learning can be observed online during the first M1 a-tDCS session [34], while online potentiating effects of M1 a-tDCS on implicit motor learning in older adults become significant only when more extended practice is allowed. When tested in young adults, however, implicit motor skills acquisition is significantly facilitated by a single session of combined M1-a-tDCS/SRT task [19]. One possible explanation for age-associated delayed effects of M1 a-tDCS on implicit motor learning could be related to the known decline of neuronal plasticity mechanism associated with aging [5, 29, 30]. Indeed, online effects of M1 a-tDCS on motor learning have been shown to depend on the activation of cerebral plasticity mechanisms in order to modify the level of excitability of the stimulated neurons [2–5]. Accordingly, a recent study showed that the largest increase in M1 corticospinal excitability was delayed in older adults and occurred 30 minutes after a-tDCS stimulation, while it was immediately after stimulation for the young group [47]. In that study, the extent of increases in M1 cortical excitability induced by a-tDCS, however, did not vary reliably between young and older adults. These findings suggest that TDCS-induced plastic changes are delayed as a result of healthy aging but that overall efficacy of M1 plasticity mechanisms is unchanged despite aging [47]. In another study conducted in young adults that applied a-tDCS to the prefrontal cortex during multitasking performance, a-tDCS was found to have delayed benefits that reflected an enhanced rate of learning [48]. Although further research is required to get a better grasp of the mechanism of action of such delayed effects, findings from the latter study indicate that a-tDCS may have delayed effects on learning across all age groups. Alternatively, a recent review identified anatomical and functional changes in the striatum as a chief neural correlate of age-related changes in motor sequence learning [49]. Knowing that anodal tDCS over M1 modulates elements of the corticostriatal functional motor circuit [50], it would appear plausible that M1 a-tDCS stimulation parameters at session 1 were not optimal to modulate striatal activity in a way that facilitated motor sequence learning in older adults.

Expectedly, both groups showed a clear, linear improvement in motor performance, that is, a global day-to-day decrease of RT in both sequence and random blocks. It has been shown that the mere repetition of a motor task over multiple days is sufficient to induce performance improvements through practice and consolidation processes [51], regardless of age [52]. When we averaged RTs of sequence blocks from sessions 1 and 5, aging adults in the a-tDCS group were significantly faster than sham counterparts. However, within-group RT improvements from session 1 to session 5 did not significantly differ across groups, suggesting that M1 a-tDCS beneficial effects over five consecutive days were restricted to sequence-specific learning. This finding is consistent with the known beneficial effects of M1 a-tDCS on practice-dependent, sequence-specific motor learning [19].

Interestingly, offline consolidation of explicit motor skills learning in response to M1 a-tDCS was recently found to be time-dependent as opposed to sleep-dependent [46]. Indeed, the mere passage of time, but not overnight sleep, was found to regulate offline skill gains induced by M1 a-tDCS. Furthermore, the latter study also showed that M1 a-tDCS influenced consolidation only when combined with concurrent motor training. In the present study, quality of sleep for the five nights that preceded training sessions was equivalent across groups, which limited potential sleep-related contaminating effects on offline consolidation.

The demonstration of the efficacy of a multisession combined M1 a-tDCS/SRT task protocol to achieve reproducible improvements in the ability to learn new sequences over five consecutive training days in an aging population is of considerable clinical interest considering that neuronal plasticity as well as skill acquisition capacities has repeatedly been shown to decline with age [34, 53, 54]. This is crucially important as reduced skill acquisition has been associated with early age-related functional decline [55]. In addition, an increased number of older adults with comorbidities affecting the motor system (e.g., stroke, TBI) could benefit from optimized rehabilitation to improve motor function. Multisessions a-tDCS concomitant with motor training could reveal to be useful, particularly in the context of intensive rehabilitation programs during which activities involving motor system function are repeated over several days. Future studies should investigate whether such motor skill refinement facilitated by multisession M1 a-tDCS protocols could persist beyond the acute postintervention phase in older adults.

While this study provides new, exciting findings on the added value of combined, multisession M1 a-tDCS protocols with the aging population, this study is not without limitations. As always, the small sample size found herein restricts the generalization of our study findings to the general population. It is also of interest to note the relatively high level of education of our study sample relative to the general population.

## 5. Conclusion

The current findings reveal that, relative to sham tDCS, the application of five consecutive, daily 20-minute sessions of M1 a-tDCS concomitant with the execution of a SRT task significantly enhances implicit motor learning in the aging brain. Indeed, our study findings show that aging individuals who were assigned to the a-tDCS protocol achieved reproducible improvements in their ability to learn new sequences over five consecutive training days, whereas a plateau was reached after only the first training session in age-equivalent controls who performed the same sequences without a-tDCS stimulation. In short, the addition of a-tDCS to motor training allows for further refinement of the skill to learn new sequences over 5 consecutive days. These findings raise into prominence the potential utility of multisession anodal TDCS protocols in combination with training to help prevent/alleviate age-associated functional decline.

## Figures and Tables

**Table 1:** Groups.

	Anodal	Sham	*t*	*P*
*N*	12	11	—	—
Male/female	6/6	5/6	.048	.827
Age	61.25 ± 5.08	60.73 ± 5.82	.269	.791
Education	17.79 ± 2.31	17.45 ± 2.77	.915	.471
BDI score	2.42 ± 2.97	3.36 ± 3.44	1.39	.179
MMSE score	29.33 ± 0.98	29.36 ± 0.81	−.080	.937

Mean ± standard deviation. Gender differences across groups were tested using a nonparametric chi-square test used to test statistical significance.

**Table 2:** Sleep quality.

	Anodal	Sham	*t*	*P*
Session 1	24.08 ± 4.94	22.77 ± 5.14	.739	.619
Session 2	23.70 ± 5.57	22.43 ± 4.35	.338	.607
Session 3	25.03 ± 5.49	23.83 ± 4.43	.650	.557
Session 4	24.65 ± 5.08	23.20 ± 4.63	.972	.713
Session 5	24.79 ± 4.22	23.35 ± 4.75	.765	.769

Mean ± standard deviation.

**Table 3:** Response accuracy (percentage of correct responses).

		Anodal	Sham	*t*	*P*
Sequence	Session 1	97.33 ± 1.68	97.85 ± 1.15	−0.51	.615
	Session 2	97.19 ± 1.86	96.71 ± 1.87	0.37	.713
	Session 3	97.24 ± 2.24	97.88 ± 1.00	−0.52	.605
	Session 4	97.32 ± 1.91	97.55 ± 1.44	−0.19	.851
	Session 5	97.61 ± 1.62	97.58 ± 1.87	0.02	.982

Random	Session 1	96.19 ± 1.97	96.48 ± 1.37	−0.24	.810
	Session 2	96.33 ± 2.36	96.79 ± 1.88	−0.30	.764
	Session 3	96.17 ± 2.72	97.39 ± 1.10	−0.84	.412
	Session 4	96.00 ± 2.90	97.24 ± 0.78	−0.83	.418
	Session 5	97.00 ± 1.82	97.12 ± 2.45	−0.08	.936

Mean ± standard deviation.
